# Screening parameters for diagnosing primary aldosteronism in patients with moderate to severe obstructive sleep apnea hypopnea syndrome and resistant hypertension

**DOI:** 10.3389/fcvm.2024.1383567

**Published:** 2024-04-24

**Authors:** Dien Yan, Xiaofan Zou, Xiao Li, Qiao Zeng, Hongbing He, Jianping Guo, Yulan Wang, Huanhuan Zheng, Jinxiang Fu, Meili Wang, Danping Peng, Xiaozi Zhou, Xian Luo, Jiahua Luo, Shaofen Li, Jinping Liu, Pingsheng Hu, Yunfeng Shen

**Affiliations:** ^1^ Department of Endocrinology and Metabolism, the Second Affiliated Hospital of Nanchang University, Nanchang, Jiangxi, China; ^2^ Department of Endocrinology, Ji'an Central Hospital, Ji'an, Jiangxi, China; ^3^ Department of Respiratory and Critical Care Medicine, Ji'an Central Hospital, Ji'an, Jiangxi, China; ^4^ Department of Obstetrics and Gynecology, Ji'an First People’s Hospital, Ji'an, Jiangxi, China; ^5^ School of Nursing, Ji'an College, Ji’an, Jiangxi, China; ^6^ Department of Neurology, Ji'an Central Hospital, Ji'an, Jiangxi, China; ^7^ Department of Laboratory, Ji'an Central Hospital, Ji'an, Jiangxi, China

**Keywords:** obstructive sleep apnea, resistant hypertension, primary aldosteronism, aldosterone-renin ratio, plasma aldosterone concentration

## Abstract

**Background:**

Patients with obstructive sleep apnea hypopnea syndrome (OSAHS) combined with resistant hypertension (RH) have a high risk of developing primary aldosteronism (PA). This study investigated the aldosterone-renin ratio (ARR), plasma aldosterone concentration (PAC), and plasma renin activity (PRA) to determine the optimal cutoff values for PA diagnosis in patients with OSAHS combined with RH.

**Methods:**

Patients diagnosed with moderate and severe OSAHS combined with RH were recruited from the inpatient clinic of the Department of Endocrinology at Ji'an Central Hospital between October 2020 and April 2023. The included patients were divided into PA and no-PA groups. Diagnostic accuracy measures were calculated for each group, and receiver operating characteristic (ROC) curves were generated.

**Results:**

A total of 241 patients were included, of which 103 had positive ARR screening results in the diagnostic accuracy analysis and 66 were diagnosed with PA. PAC and ARR showed moderate predictive capacity for PA, with area under the curve (AUC) values of 0.66 [95% confidence interval (CI): 0.55–0.77] and 0.72 (95% CI: 0.63–0.82), respectively, while PRA exhibited a limited predictive capacity (AUC = 0.51, 95% CI: 0.40–0.63). Using 45 as the optimal cutoff value for ARR, the sensitivity was 86% and the specificity was 52%. The optimal cutoff value for PAC was 17, with a sensitivity of 78% and a specificity of 55%. Notably, in patients with severe OSAHS, ARR at screening demonstrated significant predictive value for PA, with an AUC of 0.84 (95% CI: 0.72–0.96), a sensitivity of 85%, and a specificity of 76%. Conversely, in patients with moderate OSAHS, only ARR demonstrated significant predictive value for PA diagnosis, while PAC did not demonstrate notable diagnostic value.

**Conclusion:**

ARR and PAC are initial screening tools for PA, facilitating early detection, particularly in low-resource settings. In patients with OSAHS and RH, the ARR and PAC thresholds for PA diagnosis may require more stringent adjustment.

## 1 Introduction

Obstructive sleep apnea hypopnea syndrome (OSAHS) is a common sleep respiratory disorder characterized by apnea, intermittent hypoxia, and sleep fragmentation due to recurrent partial or total collapse of the upper airway during sleep (1). The population prevalence of OSAHS ranges from 9% to 38% (2), and its diagnosis relies mainly on polysomnography.

OSAHS is recognized as a contributing factor to hypertension and holds a pivotal role in cases of resistant hypertension (RH) (3). A substantial prevalence of OSAHS exists among individuals with RH, with suggestions attributing the hormone aldosterone as a potential contributor to the resistance observed in this population (4). OSAHS-induced intermittent hypoxia leads to increased sympathetic activity, resulting in activation of the renin-angiotensin-aldosterone system (RAAS), which increases the risk of hypertension (3). Primary aldosteronism (PA) is a disorder in which the zona glomerulosa of the adrenal cortex abnormally secretes aldosterone hormone, resulting in elevated plasma aldosterone concentrations and decreased plasma renin activity. It is characterized by hypertension and hypokalemia (5). PA is considered a common cause of secondary RH (6), the prevalence of which ranges from 3% to 13% in the overall population (7, 8).

Previous studies have suggested that the co-morbidity of OSAHS and PA may mutually affect and exacerbate each other (9, 10).The prevalence of PA in the OSAHS population is as high as 34% (11), and the prevalence of OSAHS in the PA population is even higher at 78.1% (12). On one hand, excessive aldosterone secretion in patients with PA leads to water and sodium retention, causing edema of pharyngeal wall tissues and aggravating the severity of OSAHS (13). On the other hand, intermittent nocturnal hypoxia in patients with OSAHS may increase the activity of RAAS, which may lead to the occurrence and development of PA (9).

Since patients with RH and OSAHS are at high risk of developing PA, accurate PA diagnosis is crucial for targeted therapy, which can subsequently reduce the associated cardiovascular risk (14). Nevertheless, the diagnosis of PA involves a multifaceted process encompassing screening, diagnostic assessments, and subtyping, posing challenges due to its intricacy and time-intensive nature. Many healthcare facilities in China lack the necessary resources to complete this comprehensive process, resulting in delays in timely diagnosis and intervention. Therefore, the initial screening test assumes paramount importance in identifying individuals at risk of PA. However, existing guidelines do not offer a definitive cutoff value for the corresponding screening indicator for the diagnosis of PA in patients presenting with both RH and OSAHS. Therefore, this study aimed to explore the optimal cutoff values of PA-related diagnostic screening parameters, including the plasma aldosterone concentration (PAC), plasma renin activity (PRA), and aldosterone-renin ratio (ARR) in patients with moderate and severe OSAHS combined with RH.

## 2 Methods

### 2.1 Study design and population selection

**Figure 1:**
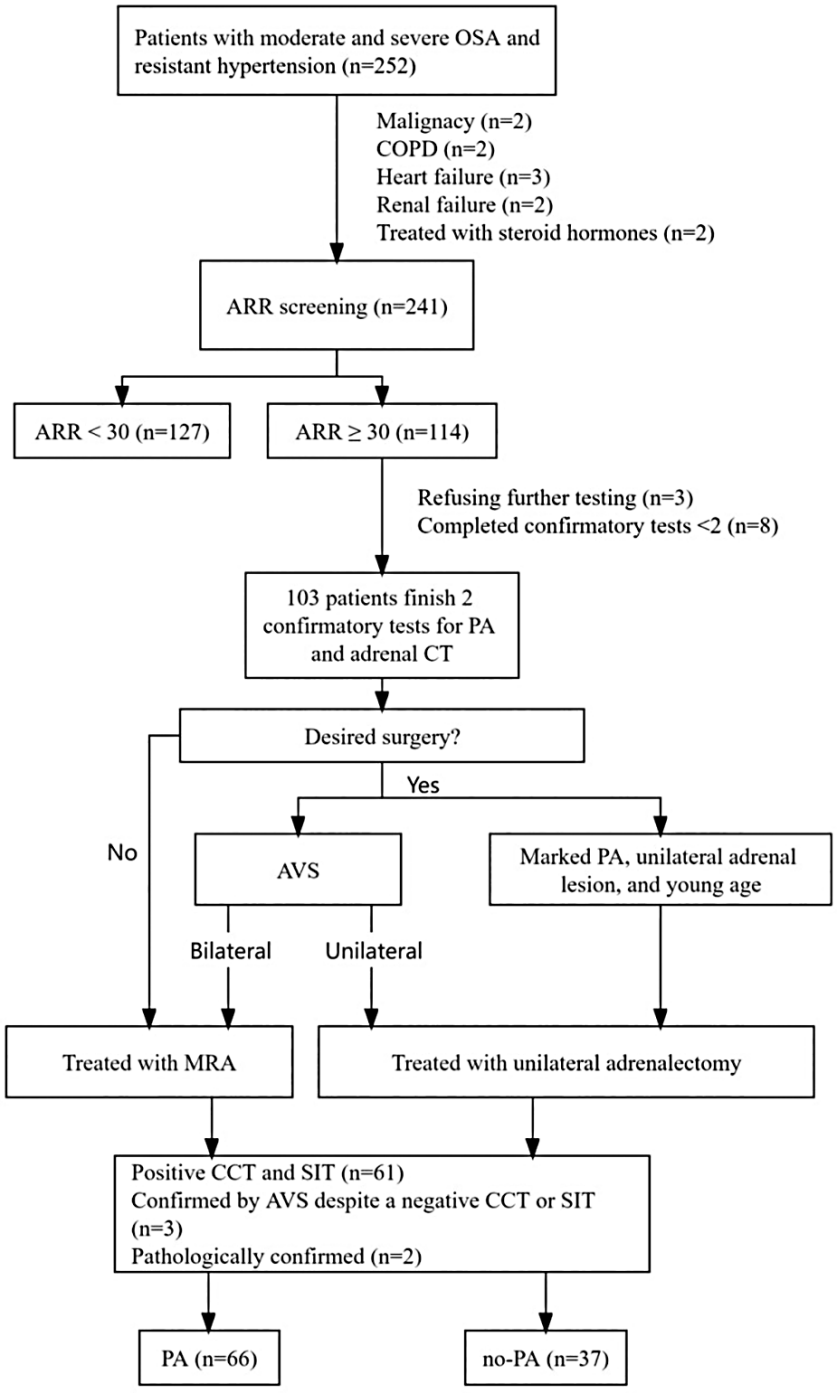
Flowchart of inclusion and exclusion criteria of study participants. ARR, plasma aldosterone-renin ratio; AVS, adrenal venous sampling; CCT, captopril challenge test; COPD, chronic obstructive pulmonary disease; MRA, mineralocorticoid receptor agonist; OSA, obstructive sleep apnea; PA, primary aldosteronism; SIT, saline infusion test.

This cross-sectional observational study was conducted in the inpatient clinic of the Department of Endocrinology at Ji'an Central Hospital. From October 2020 to April 2023, a total of 252 patients with moderate and severe OSAHS combined with RH were recruited (Figure 1). All participants provided written informed consent, and the study was approved by the Ethics Committee of the Ji'an Central Hospital (No. 063056).

### 2.2 Defining OSAHS

Sleep apnea was assessed with validated cardiorespiratory polygraphy with at least eight standard channels, recording oronasal flow, snoring, thoracic and abdominal respiratory movements, finger pulse oximetry, body position, and electrocardiogram (15). Apnea-hypopnea index (AHI), oxygen desaturation index, mean blood oxygen saturation, and the lowest blood oxygen saturation were collected. Apnea was defined as a reduction of oronasal flow of ≥90% for at least 10 s. Hypopnea was defined as a ≥30% reduction in oronasal flow for at least 10 s, associated with oxygen desaturation of ≥3% or arousal (15). AHI was defined as the total number of apneas plus hypopneas per hour of recording time. OSAHS was defined as AHI ≥5 and classified as mild (AHI ≥5 to <15), moderate (AHI ≥15 to <30), or severe (AHI ≥30) (16).

#### 2.2.1 Defining RH and blood pressure measurement

RH was a common clinical condition of inadequate blood pressure control, mainly characterized by blood pressure remaining above target levels despite the administration of three synergistic antihypertensive medications including thiazide diuretics, at tolerable and sufficient doses for at least 4 weeks, alongside lifestyle modifications (6). Before blood pressure measurement, the patients were asked to rest quietly for at least 5 min in a quiet, comfortable environment. The individual should sit in a comfortable chair with their back supported and their arm positioned at heart level. Upper arm medical electronic blood pressure monitor (Omron HEM-7132) was used to measure blood pressure. When measuring blood pressure, the measurement should be repeated 1–2 min apart, and the average of the two readings should be recorded. If the difference between the two readings of SBP or DBP was more than 5 mmHg, it should be measured again and the average of the three readings should be recorded.

#### 2.2.2 Exclusion criteria

The exclusion criteria were as follows: terminal malignant tumor; chronic obstructive pulmonary disease; heart failure classified as New York Heart Association class III or IV; chronic kidney disease with an estimated glomerular filtration rate <30 ml/min/1.73 m^2^.

### 2.3 Screening and confirmatory tests

For the screening test and the confirmatory tests, antihypertensive medication was withheld or changed according to the guideline (17). The screening and diagnosis of PA were conducted under standardized conditions for all patients: diuretic treatment was withheld for at least 4 weeks; β-blockers, angiotensin-converting enzyme inhibitors, angiotensin-II receptor blockers, and dihydropyridine calcium antagonists were discontinued for at least 2 weeks; patients with uncontrolled hypertension were only treated with *α*-blockers or non-dihydropyridine calcium antagonists; participants' sodium intake was limited; potassium levels were corrected to normal in patients with hypokalemia. Before screening for PA, patients should stay in a non-recumbent position (sitting, standing, or walking) for at least 2 h after getting up in the morning, and then sit still for 5–15 min before taking blood. The patient should rest in a recumbent position for at least one hour before saline infusion test (SIT) and should remain recumbent throughout SIT. Patients should sit and rest for at least 1 h before captopril challenge test (CCT) and remain seated throughout the CCT (17, 18).

#### 2.3.1 Screening tests

For screening, samples for PRA and PAC were collected after patients had maintained a supine position for at least 8 h and an upright position for at least 2 h. The aldosterone-renin ratio (ARR), calculated as aldosterone (ng/dl) divided by renin (ng/ml/h), was determined. A positive screening test result was defined as ARR ≥30 ng/dl: ng/ml/h (equivalent to 3.7 ng·dl^−1^/mIU·L^−1^) (17). Patients with an ARR ≥30 ng/dl: ng/ml/h underwent further confirmatory testing (captopril challenge test [CCT] and saline infusion test [SIT]) conducted on two separate days, along with an adrenal CT scan.

#### 2.3.2 Confirmatory tests

For SIT, patients maintained a recumbent position for at least 1 h before and during the infusion of 2l of 0.9% saline over 4 h, starting at 8:00 to 9:00 am. Blood samples for PRA, PAC, cortisol, and plasma potassium were drawn at immediately after the start of the infusion and after 4 h. Patients remained fasted during the test, with their blood pressure and heart rate closely monitored. Post-infusion plasma aldosterone levels <5 ng/dl make the diagnosis of PA unlikely, while levels >10 ng/dl are indicative of very probable PA (17).

For CCT, patients received 50 mg captopril orally between 8:00 and 9:00 a.m. after sitting or standing for at least 1 h. Blood samples were drawn for the measurement of PRA and PAC at immediately after the start of the infusion and 2 h after taking captopril, with the patient remaining seated during this period.

In individuals without PA, captopril typically suppresses PAC by >30%. However, in PA patients, PAC remains elevated despite captopril administration, while PRA remains suppressed (17).

#### 2.3.3 Further tests and treatments

Patients willing to undergo adrenalectomy were subjected to adrenal venous sampling (AVS), and the criteria used to determine the lateralization of aldosterone hypersecretion were established according to guideline (17).

Patients confirmed to have lateralization of aldosterone secretion through AVS, as well as younger patients (aged < 35) with significant aldosterone excess and unilateral adrenal lesions, proceeded with unilateral adrenalectomy accompanied by pathological biopsy. Meanwhile, patients confirmed to have bilateral aldosterone production through AVS or those unwilling to undergo adrenalectomy received mineralocorticoid receptor antagonist.

### 2.4 Diagnostic criteria

The diagnosis of PA was established based on the following criteria: (1) both confirmatory tests (CCT/SIT) yielded positive results; (2) in cases where CCT and/or SIT results were negative, AVS examination confirmed lateralization of aldosterone secretion. If only one of the CCT and SIT was positive, the diagnosis of PA could be established if the AVS confirmed unilateral or bilateral excessive secretion of aldosterone. The operating methods and evaluation criteria of AVS are provided in the supplementary materials; (3) a pathological biopsy taken during adrenalectomy confirmed the diagnosis of PA.

### 2.5 Statistical analysis

Normally distributed variables were presented as the mean ± standard deviation (SD); variables with skewed distributions were expressed as the median (quartile range); and categorical variables were described as a percentage. To compare patient attributes between the OSAHS-RH-no PA and OSAHS-RH-PA groups, a dual methodology of analysis was employed: independent-samples *t*-test and Mann-Whitney *U* test for quantitative variables and Chi-square test for categorical variables. To assess the diagnostic accuracy of selected diagnostic variables, parameters including sensitivity, specificity, positive predictive value, negative predictive value, positive likelihood ratio, and negative likelihood ratio were calculated. The receiver operating characteristic (ROC) curves were generated, and the areas under the curves (AUC) of different parameters were calculated. The statistical tests were performed using a two-sided approach, and a significance level of *P* < 0.05 was considered statistically significant. All data analyses were conducted using R software (R-project®; R Foundation for Statistical Computing, Vienna, Austria, version 4.2.1) and STATA 16 (Stata Corp. 2019, Stata Statistical Software: version 16, Stata Corp LLC).

## 3 Result

### 3.1 Baseline characteristics of the study population

A total of 252 patients with moderate and severe OSAHS and RH were enrolled, with 11 patients excluded because of malignancy (*n* = 2), chronic obstructive pulmonary disease (*n* = 2), heart failure (*n* = 3), renal failure (*n* = 2), and receiving steroid hormones (*n* = 2). ARR screening was completed by 241 patients. According to the protocol, 114 patients were eligible for confirmatory tests after ARR screening, among whom 11 patients were excluded: 3 refused further testing, and 8 did not complete both confirmatory tests. Finally, 103 patients completed both confirmatory tests for PA as well as adrenal CT, and 66 patients were diagnosed with PA: 61 patients with positive CCT and SIT, 3 patients were confirmed by AVS despite only one positive CCT or SIT, and 2 patients were confirmed pathologically (two young patients [<35 years old] with spontaneous hypokalemia, significant aldosterone secretion [PAC >20 ng/dl] and unilateral adrenal adenoma on CT scan underwent surgical treatment and were pathologically diagnosed with PA).

A total of 103 participants were included in the study, with a mean age of 50.31 ± 13.09 years. Out of these patients, 41 (39.8%) were female. The patients were divided into two groups based on their PA diagnosis: the OSAHS-RH-no PA group (*n* = 37) and the OSAHS-RH-PA group (*n* = 66). Table 1 presents an overview of the baseline characteristics of the included patients. Patients in the OSAHS-RH-PA group were associated with higher levels of triglycerides, total cholesterol, 24-h urinary potassium, plasma creatinine, PAC at screening, ARR at screening, and a higher rate of adrenal nodules, while serum potassium and PRA at screening were lower compared to those in the OSAHS-RH-no PA group (all *p* < 0.05). There were no significant differences observed in other variables between the two groups, including age, gender, duration of hypertension, diabetes, body mass index, smoking, systolic blood pressure, diastolic blood pressure, high-density lipoprotein cholesterol, AHI, oxygen desaturation index, mean blood oxygen saturation, and lowest blood oxygen saturation (all *p* > 0.05).

**Table 1:** Comparison of baseline characteristics between OSAHS-RH-no PA and OSAHS-RH-PA groups.

Variables	OSAHS-RH-no PA	OSAHS-RH-PA	*P* value
	*n* = 37	*n* = 66	
Age, year	49.65 ± 13.13	50.68 ± 13.16	0.703
Duration of hypertension	13.68 ± 11.94	12.94 ± 10.01	0.739
Female, *n* (%)	18 (48.65%)	23 (34.85%)	0.171
Diabetes, *n* (%)	9 (24.32%)	20 (30.30%)	0.517
BMI, kg/m^2^	29.66 ± 4.93	31.04 ± 3.49	0.101
SBP, mm Hg	145.35 ± 13.06	143.97 ± 15.38	0.646
DBP, mm Hg	90.22 ± 12.29	86.85 ± 12.22	0.184
Triglyceride, mmol/L	1.42 (1.01, 1.84)	2.01 (1.37, 3.46)	0.001
Total cholesterol, mmol/L	4.56 ± 1.11	5.22 ± 1.32	0.012
HDL-c, mmol/L	1.01 ± 0.27	1.09 ± 0.33	0.231
Serum K^+^, mmol/L	3.97 ± 0.47	3.57 ± 0.46	<0.001
24 h urinary K^+^, mmol/L	35.57 ± 14.53	61.42 ± 25.58	<0.001
Plasma creatinine, μmol/L	76.19 ± 15.99	87.20 ± 17.15	0.002
AHI, times/h	30.49 ± 9.75	32.59 ± 8.83	0.266
ODI, times/h	17.65 ± 6.58	18.82 ± 5.93	0.358
MSaO_2_, %	87.08 ± 3.07	86.71 ± 3.39	0.585
LSaO_2_, %	82.43 ± 2.96	82.17 ± 3.32	0.686
PAC at screening, ng/dl	15.11 ± 3.63	17.17 ± 4.31	0.016
PRA at screening, ng/ml/h	1.12 ± 0.44	0.89 ± 0.32	0.003
ARR at screening, ng/dl: ng/ml/h	39.44 ± 5.45	46.12 ± 9.72	<0.001
Adrenal nodular, *n* (%)	11 (29.73%)	63 (95.45%)	<0.001

AHI, apnea-hypopnea index; ARR, plasma aldosterone-renin ratio; BMI, body mass index; DBP, diastolic blood pressure; HDL-c, high-density lipoprotein cholesterol; LSaO_2_, lowest blood oxygen saturation; MSaO_2_, mean blood oxygen saturation; ODI, oxygen desaturation index; OSAHS, obstructive sleep apnea hypopnea syndrome; PA, primary aldosteronism; PAC, plasma aldosterone concentration; PRA, plasma renin activity; RH, resistant hypertension; SBP, systolic blood pressure.

### 3.2 Results of confirmatory tests between the OSAHS-RH-no PA and OSAHS-RH-PA groups

The PAC and the PRA were evaluated after SIT and CCT in both groups (Table 2). In SIT, patients in the OSAHS-PA group exhibited significantly higher levels of PAC and PRA at both at immediately after the start of the infusion and 4 h compared to patients in the OSAHS-no PA group. In CCT, there was no significant difference in PAC at 0 h between patients in the OSAHS-PA group and those in the OSAHS-no PA group. However, at 2 h, patients in the OSAHS-PA group had significantly higher PAC levels compared to those in the OSAHS-no PA group, and the PAC suppression percentage was significantly lower in the OSAHS-PA group compared to the OSAHS-no PA group. PRA in both groups showed no significant differences at 0 h and 2 h.

**Table 2:** The results of SIT and CCT tests in OSAHS-RH-no PA and OSAHS-RH-PA groups.

Variables of confirmatory tests	OSAHS-no PA	OSAHS-PA	*P* value
	*n* = 37	*n* = 66	
SIT			
PAC at 0 h	14.73 ± 4.51	20.71 ± 6.35	<0.001
PAC at 4 h	5.54 ± 1.96	16.55 ± 6.19	<0.001
PRA at 0 h	0.42 ± 0.13	0.50 ± 0.09	<0.001
PRA at 4 h	0.32 ± 0.12	0.38 ± 0.13	0.026
CCT			
PAC at 0 h	15.92 ± 16.96	16.56 ± 6.32	0.783
PAC at 2 h	8.72 ± 1.97	13.86 ± 5.01	<0.001
PRA at 0 h	0.49 ± 0.15	0.54 ± 0.13	0.101
PRA at 2 h	0.53 ± 0.18	0.58 ± 0.17	0.208
PAC-SP at 2 h	36.22 ± 9.60	15.73 ± 5.12	<0.001

CCT, captopril challenge test; OSAHS, obstructive sleep apnea hypopnea syndrome; PA, primary aldosteronism; PAC, plasma aldosterone concentration; PAC-SP, plasma aldosterone concentration suppression percentage; PRA, plasma renin activity; SIT, saline infusion test.

### 3.3 The diagnosis value of PA among different indicators by ROC graph curves

**Figure 2:**
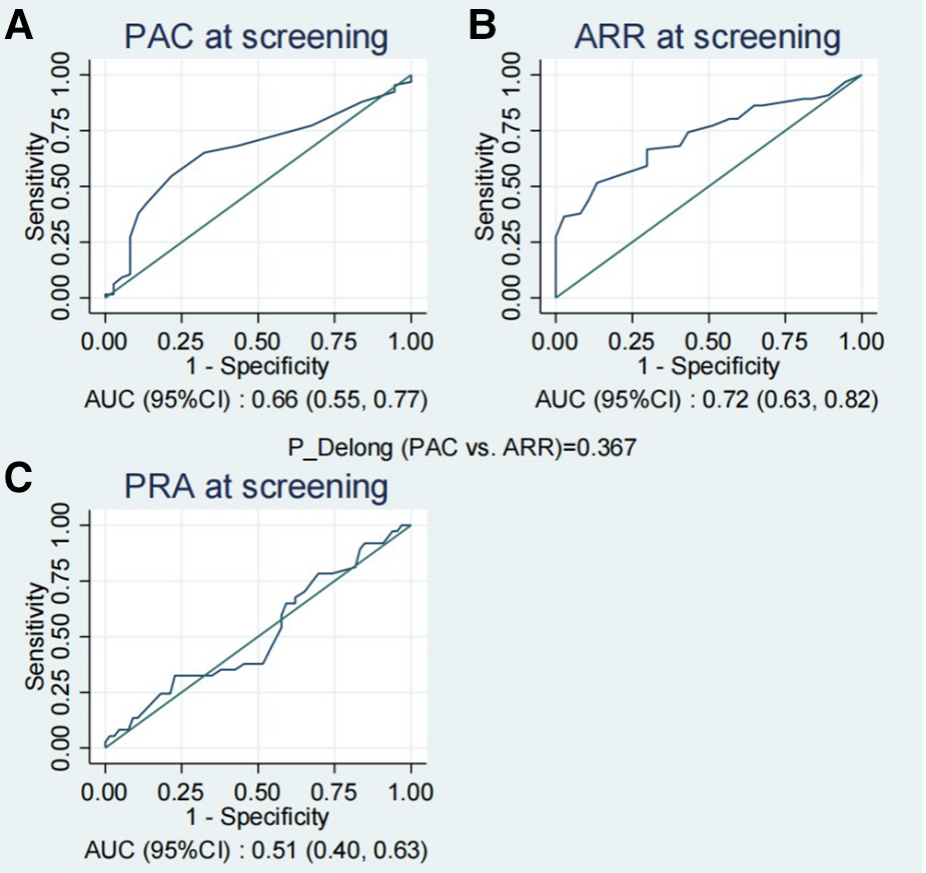
The ROC curves of PAC (**A**), ARR (**B**) and PRA (**C**) at screening for the prediction of PA. ARR, plasma aldosterone-renin ratio; PAC, plasma aldosterone concentration; PA, primary aldosteronism; PRA, plasma renin activity; ROC, receiver operating characteristic.

The ROC graph curves presented in Figure 2 demonstrated that PAC and ARR at screening possessed a moderate predictive capacity for the prediction of PA, with AUC values of 0.66 (95% CI: 0.55–0.77) and 0.72 (95% CI: 0.63–0.82), respectively, while PRA exhibited limited predictive capacity with an AUC of 0.51 (95% CI: 0.40–0.63). Moreover, there were no statistically significant differences observed in PAC and ARR at screening (Delong test *p* = 0.367 for PAC vs. ARR).

Table 3 provides the corresponding diagnostic performance metrics. Using 45 as the cutoff value for ARR at screening, we achieved a sensitivity of 52% and a specificity of 86%, a positive predictive value of 87%, and a negative predictive value of 50%. The optimal cutoff value for PAC was 17, with a relatively lower specificity (78%) than ARR, a sensitivity of 55%, a positive predictive value of 82%, and a negative predictive value of 49% (Table 3). Among the three indicators, ARR at screening, and PAC at screening exhibited potential for the diagnosis of PA in patients with moderate and severe OSAHS combined with RH.

**Table 3:** Predictive value of PAC and ARR at screening for the diagnosis of PA.

Diagnostic variables	Optimal cutoff values	a	b	c	d	Specificity	Sensitivity	LR+	PV+	PV-
ARR at screening	45	34	5	32	32	0.86	0.52	3.81	0.87	0.5
PAC at screening	17	36	8	30	29	0.78	0.55	2.52	0.82	0.49

a: true positive; b: false positive; c: false negative; d: true negative.

ARR, plasma aldosterone-renin ratio; LR+, positive likelihood ratio; LR-, negative likelihood ratio; PA, primary aldosteronism; PAC, plasma aldosterone concentration; PV+, positive predict value; PV-, negative predict value.

### 3.4 The diagnosis value of PAC and ARR at screening for PA according to the degree of OSAHS

**Figure 3:**
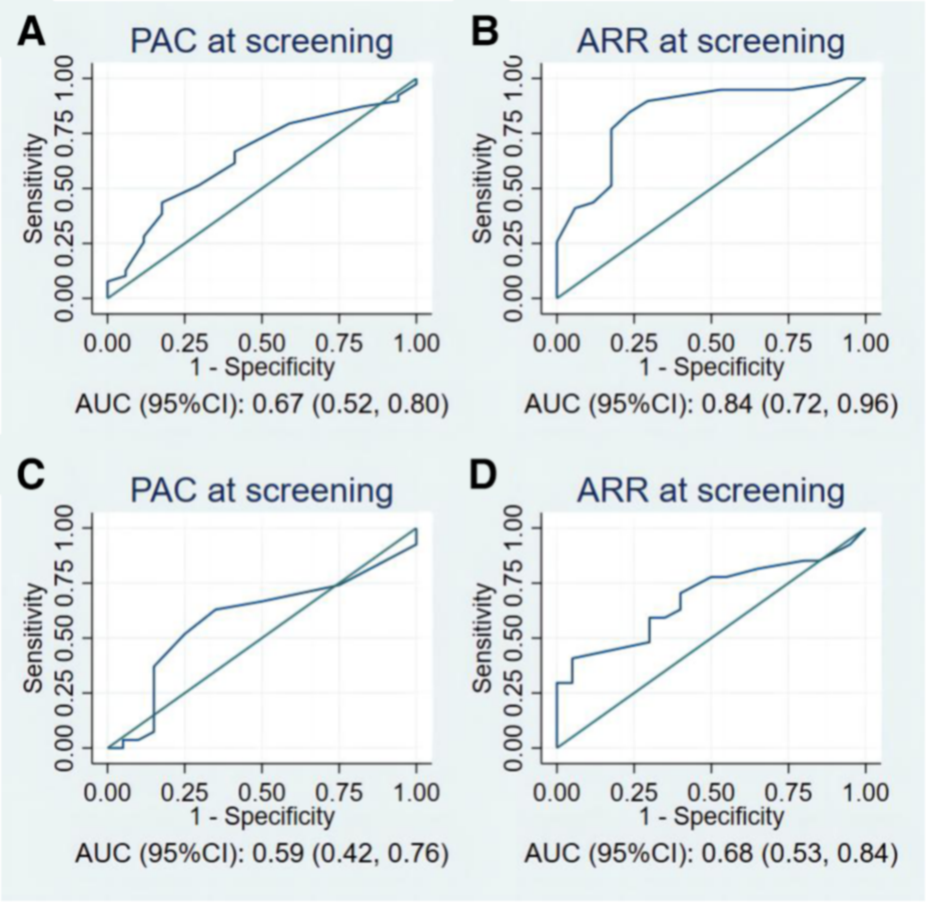
The ROC curves of PAC and ARR at screening for the prediction of PA in (**A**,**B**) patients with severe OSAHS, and (**C**,**D**) patients with moderate OSAHS. PAC, plasma aldosterone concentration; ARR, plasma aldosterone-renin ratio; ROC, receiver operating characteristic; PA, primary aldosteronism; OSAHS, obstructive sleep apnea hypopnea syndrome.

Figure 3 and Table 4 present the value of PAC and ARR at the screening stage for the diagnosis of PA in patients with moderate and severe OSA. In the patients with severe OSAHS, PAC at screening and ARR at screening held diagnostic value for PA. Particularly, ARR has a significant predictive value with an AUC of 0.84 (95% CI: 0.72–0.96) (Figure 3B). Using 42 as the cutoff value for ARR at screening, we achieved a sensitivity of 85% and a specificity of 76%, a positive predictive value of 89%, and a negative predictive value of 68%. In patients with moderate OSAHS, ARR at screening held diagnostic value for PA, whereas PAC did not.

**Table 4:** Predictive value of PAC at screening and ARR at screening for the diagnosis of PA according to OSAHS.

Diagnostic variables	Optimal cutoff values	a	b	c	d	Specificity	Sensitivity	LR+	LR-	PV+	PV-
Patients with severe OSAHS											
ARR at screening	42	33	4	6	13	0.76	0.85	3.60	0.20	0.89	0.68
PAC at screening	18	17	3	22	14	0.82	0.43	2.47	0.69	0.85	0.39
Patients with moderate OSAHS											
ARR at screening	45	11	1	16	19	0.95	0.41	8.15	0.62	0.92	0.54
PAC at screening	16	17	7	10	13	0.65	0.63	1.80	0.57	0.71	0.57

a: true positive; b: false positive; c: false negative; d: true negative.

ARR, plasma aldosterone-renin ratio; LR+, positive likelihood ratio; LR-, negative likelihood ratio; OSAHS, obstructive sleep apnea hypopnea syndrome; PA, primary aldosteronism; PAC, plasma aldosterone concentration; PV+, positive predict value; PV-, negative predict value.

## 4 Discussion

This study investigated the diagnostic value of common screening indicators, including PRA, PAC, and ARR, for identifying PA among patients with moderate and severe OSAHS combined with RH. It is important to note that while these indicators can be utilized in settings with limited resources, they are not substitutes for definitive diagnostic tests. Among the 241 patients included in the study, 66 were diagnosed with PA. We found that ARR, across all patients, and PAC, especially in those with severe OSAHS, displayed moderate predictive utility for PA. In contrast, PRA exhibited limited predictive capacity for PA diagnosis, lacking sufficient diagnostic value compared to ARR and PAC. Notably, in the subgroup of patients with severe OSAHS, the screening efficacy of PAC and ARR was particularly significant. Early identification of PA using these indicators can lead to prompt intervention and improve patient outcomes. While these indicators can streamline the initial assessment process in patients at higher risk of PA and potentially reduce costs, the definitive diagnosis should rely on more conclusive tests.

Recently, there has been an observed high prevalence of PA in individuals with OSAHS and vice versa (19). PA has been identified in 2.6%–12.7% of patients with hypertension (20). Among individuals with both OSA and hypertension, the prevalence of PA was reported to be at least 5%, reaching as high as 36% in cases of RH combined with probable OSAHS (13, 21, 22). PA appears to be more prevalent among individuals with OSAHS and hypertension when compared to the broader hypertensive population. In a cohort of 114 subjects diagnosed with RH, 72 were identified as at high risk of sleep apnea according to the Berlin questionnaire. Notably, those at high risk of sleep apnea exhibited a higher likelihood of being diagnosed with PA, with a prevalence of 36% compared to 19% among those classified as low risk (21). Moreover, in a study by Di Murro et al. wherein both PA and OSAHS were conclusively diagnosed, it was observed that patients with OSAHS (*n* = 53) had a significantly greater prevalence of PA (34%) in comparison to those without OSAHS (9.2%, *n* = 272) (11). Our study found that the prevalence of PA in patients with OSAHS and RH was approximately 27%, which is slightly lower than the previously reported rates (36%) (11), possibly due to the more stringent diagnostic criteria used in our study.

Studies have suggested that the co-morbidity of OSAHS and PA can mutually affect and exacerbate each other (9, 10). Possible mechanisms include the following factors. In patients with PA and OSAHS, aldosterone excess-induced volume overload contributes to OSAHS pathogenesis by promoting sodium-water reabsorption and elevating overnight fluid shifting to the neck in the supine position, resulting in pharyngeal edema and upper airway obstruction (19). Aldosterone excess could also damage the taste sensitivity of sodium chloride and promote increased salt intake, further facilitating water-sodium retention (23). Additionally, it has been demonstrated that OSAHS severity was strongly correlated with an overnight reduction of leg fluid volume and calf circumference (24). Conversely, intermittent hypoxia caused by OSAHS might, in turn, exacerbate RAAS activation. Besides, significantly increased secretion of cortisol in patients with PA, independent of PA subtype or adenoma tissue genotypes, could also increase the risk of OSAHS (25, 26).

Studies have indicated a higher incidence of complications of the cardiovascular system, brain, and kidneys in patients with PA compared to those with essential hypertension (27). Therefore, in individuals with OSAHS and RH, early screening and prompt initiation of appropriate treatment for PA are crucial for preventing target organ damage and improving prognosis. Accurate PA diagnosis enables precise therapy, resulting in reduced cardiovascular risk (14). Clinical studies showed that aldosterone blockade in patients with OSAHS and PA reduced not only PAC and blood pressure but also AHI and neck girth (28–30). Thus, the initial screening test holds significant importance in identifying individuals at risk. While the ARR is considered the most reliable screening method for PA, current guidelines recommend its use as a detection test only, with patients testing positive advised to undergo further confirmatory tests for a definitive diagnosis, including the oral sodium loading test, SIT, CCT, and the fludrocortisone suppression test (17). However, due to the limitations of most studies involving patients with hypertension evaluating the diagnostic performance of these confirmatory tests, such as retrospective design, relatively small patient size, and selection bias, there is no identified gold standard confirmatory test for PA (17). Additionally, due to their intricate and time-consuming nature, confirmatory tests often require patients to be hospitalized and assisted by medical personnel, adding to the inconvenience of their clinical application. For example, although some researchers consider the fludrocortisone suppression test the most reliable method for confirming PA, it is impractical due to its complexity, difficulty in execution, relatively high cost, and prolonged hospitalization required (31). On the other hand, SIT, while being one of the most widely used confirmatory tests for PA, has a test duration exceeding 4 h and may lead to various adverse effects on cardiovascular function and/or electrolyte metabolism, limiting its applicability in routine clinical practice (27). To our knowledge, this study is the first to examine the potential of ARR and PAC at the screening stage for identifying PA in patients with moderate to severe OSAHS combined with RH. Our findings suggest that ARR and PAC can serve as preliminary indicators for PA diagnosis in this specific population, where patients at high risk are commonly seen in primary and secondary care settings that may lack the resources for comprehensive PA diagnostics. Hence, adopting more aggressive cutoff values for PA screening in these settings could enable prompt initiation of appropriate therapy, potentially reducing the risk of cardiovascular events. While our results indicate that when the ARR at screening is >45 ng/dl: ng/ml/h and/or the PAC at screening is >17 ng/dl, confirmatory testing for PA might be less urgent, it is crucial to emphasize that these screening indicators should not replace confirmatory tests. Instead, they can be used to prioritize patients for further testing and treatment, especially in resource-limited scenarios such as remote areas or when patients are unable to undergo extensive testing. This strategy aims to facilitate prompt initiation of guideline-directed medical therapy for PA, mitigating time and cost burdens as well as minimizing patient risks.

This study addresses a notable research gap by providing a novel assessment of the effectiveness and diagnostic thresholds of the ARR as a screening tool for PA in patients with moderate and severe OSAHS combined with RH. The distinctiveness of our work resides in the comprehensive assessment of ARR's utility specifically within a high-risk patient population, an aspect not extensively covered in prior studies. By establishing specific diagnostic thresholds that correlate with the severity of OSAHS, our research enhances the understanding of PA screening within the context of OSAHS and RH, emphasizing the critical role of early detection and intervention. A particularly intriguing aspect of our findings is the differential ARR cutoff values identified for diagnosing PA in patients with moderate and severe OSAHS, specifically 45 for moderate OSAHS and a lower threshold of 42 for severe OSAHS. This difference suggests potential variations in underlying pathophysiological mechanisms associated with OSAHS severity. The lower cutoff value for severe OSAHS implies that patients with severe sleep apnea are at a higher risk for PA, indicating that OSAHS severity may also affect the optimal diagnostic threshold for using ARR as a screening tool. This observation emphasizes the need for a nuanced approach for PA screening in patients with OSAHS, where the severity of sleep apnea should be considered in determining the optimal ARR cutoff value for individual patients. This adjustment could enhance the specificity and sensitivity of PA screening in this high-risk population, ultimately leading to more personalized and effective diagnostic strategies. Furthermore, it underscores the importance of tailoring PA management strategies to each patient's OSAHS severity, offering a more targeted approach to treatment and potentially improving outcomes. These findings have significant implications, suggesting that future guidelines for PA screening in patients with OSAHS could benefit from incorporating severity-specific ARR cutoffs.

Several limitations should be acknowledged when interpreting the findings of our study. First, this study was a single-center, cross-sectional observational study, and inevitable bias may affect the authenticity of the results, potentially weakening their reliability. Second, due to the lack of uniformity in diagnostic protocols and assay methods for measuring the ARR in different medical centers (17), the resulting cutoff values may vary substantially, limiting the generalization of the results of this study. Multi-center research is needed to further verify our findings among a broader population. Third, in some cases, when patients had only one positive result in CCT and SIT, and expressed a strong desire for surgery, we performed adrenal vein sampling (AVS) on them. Those confirmed by AVS with bilateral or unilateral excessive secretion of aldosterone were diagnosed with primary aldosteronism (PA). However, it should be acknowledged that this diagnostic criterion carries some degree of scientific uncertainty. The AVS examination is typically used for subtype classification rather than confirming the diagnosis of PA itself. Future studies will adhere more closely to diagnostic criteria guided by clinical guidelines. Lastly, our study was conducted in a single tertiary hospital center and involved a highly selected population. Therefore, our results need to be tested in a broader population to enhance their applicability and generalizability.

## 5 Conclusion

The current study focused on patients at high risk of PA, specifically those with moderate and severe OSAHS combined with RH, and found that the prevalence of PA in this population was as high as approximately 27%. Our findings shed light on the diagnostic potential of the ARR and PAC as screening tools, with differential ARR cutoff values identified for diagnosing PA in patients with moderate and severe OSAHS (45 for moderate OSAHS and 42 for severe OSAHS). The lower ARR cutoff value for severe OSAHS suggested a higher risk of PA in this population, indicating that patients with more severe OSAHS might require earlier intervention for PA. This study contributes to existing knowledge by advocating for the nuanced application of ARR in screening practices, especially in settings where PA is prevalent among patients with OSAHS and RH. It is important to emphasize, however, that these indicators serve as preliminary aids and should not replace the confirmatory tests in PA diagnosis.

## Data Availability

The raw data supporting the conclusions of this article will be made available by the authors, without undue reservation.
